# Influences of Mg-activation on sugarcane bagasse biochar characteristics and its PNP removing potentials from contaminated water

**DOI:** 10.1038/s41598-023-46463-8

**Published:** 2023-11-06

**Authors:** Ayman H. Mansee, Doaa M. Abdelgawad, Eman H. El-Gamal, Amal M. Ebrahim, Maher E. Saleh

**Affiliations:** 1 Department of Pesticide Chemistry and Technology, Faculty of Agriculture, Alexandria University, Alexandria, Egypt; 2 Land and Water Technologies Department, Arid Lands Cultivation Research Institute (ALCRI), City of Scientific Research and Technological Applications (SRTA-City), New Burg El-Arab, Egypt; 3 Department of Soil and Water Science, Faculty of Agriculture, Alexandria University, Alexandria, Egypt

**Keywords:** Environmental sciences, Risk factors

## Abstract

Biochar as a substitute eco-friendly and low-cost adsorbent is introduced for removing* p*-nitrophenol (PNP) one of the most important chemical contaminant that recognized as the main metabolite in many pesticides and an intermediate compound in many industries. Physicochemical characteristics of sugarcane bagasse biochar (SCBB) and its Mg-activation (ASCBB) generated at 500 °C for 30 min were investigate. Batch kinetic experiment was conducted (200 mg L^−1^ PNP) to evaluate sorption efficiency of both tested biochars. To study the reaction behavior of PNP adsorption on ASCBB, solution pH and isotherm experiment of different concentrations and dosages were as investigated. The results show that ASCBB had a higher biochar yield, ash content, pH, molar ratios (H/C and O/C), surface area, pore volume, mean pore diameter, and specific and thick wall structure than SCBB. The efficiency of ASCBB to remove PNP was higher than SCBB which reached 51.98% in the first 1 min., and pH 7 achieved the optimum adsorption. Pseudo-second-order model examination exhibited well fitted to explain the adsorption results depending on R^2^ value (1.00). The adsorption isotherm results were well described by the Elovich and Freundlich models depending on the R^2^, q_m_ and n values, which means the formation of a multilayer of PNP on the ASCBB surface through the chemisorption reaction. The calculated q_m_ (144.93 mg g^−1^) of 1g L^−1^ was relatively close with experimental value (142.03 mg g^−1^). The PNP adsorption mechanism on both biochar types was electrostatic attraction, hydrogen bonding, and π-π stacking interactions, which were confirmed by studying the surface reactions before and after adsorption. Overall, the current study provided a successful waste biomass-derived biochar as a conducive alternative eco-sorbent to eliminate *p*-nitrophenol from wastewater.

## Introduction

The impact of industrialization and agriculture activities on increasing the pollutants (type and level) in the different environmental phases (water, soil, and air) is obviously clear. One of the most severe pollutants from those activities is the *p*-nitrophenol (PNP) compound. PNP has marked specifications, such as non-degradable and bio-accumulative properties and intensive toxic effects on methemoglobin formation. Such specifications oriented USEPA to list this compound as priority pollutants^1^. Moreover, the high polarization properties of PNP alert removing it from water and suggest considering it as a superior model target solute for testing the efficiencies of any suggested remediation materials and methods^2,3^.

PNP is developed from the production and application of pesticides, petrochemical synthesis, dyes production, petroleum refining, and plastics manufacturing^4^. Because it had a wide distribution among agriculture and industry, with environmental and human life risks, it alerts researchers to deal with its potential hazards. PNP is poisonous, has carcinogenic effects, and has a bioaccumulative effect, according to studies^5,6^. As a result, numerous nations have designated it as a priority contaminant. The ecology and public health would be at risk if wastewater containing PNP was directly discharged into the receiving water. According to reports, exposure to PNP may harm the central nervous system, the blood system, and key organs such as the lung, kidney, and eye^7–9^.

Different physical, chemical, and biological technologies have been used to eliminate organic pollutants from the environment including adsorption, electrolysis, advanced oxidation processes, membrane filtration, and biodegradation^10–18^. However, adsorption technology is considered as a one of the most common methods for removing pollutants from different environmental phases, such as soil, wastewater, or air. This technology proved to reliable and is an extensively used technique for removing pollutants from different environmental phases because of its simple design, easy operations, and relatively simple regeneration^19^. Many materials, including biochar, can be employed as sorbent material to remove pollutants through adsorption process; however; the most common of those materials, are carbon-rich materials.

Biochar has been considered a highly effective sorbent for organic and inorganic contaminants in water and soil, enabling it to reduce the bioavailability and toxicity of contaminants to living organisms^20–24^. Lately, biochar is extensively used to remove pollutants from air^25,26^.

Adsorption technology using biochar, among many other sorbents, has been successfully used to remove PNP and other contaminants from different environmental media. Biochar alone or even combined with other formulas has been investigated to remove contaminants. Liu et al*.*^27^ used a combination of biochar and immobilized bacteria to remove cypermethrin contamination from soil. Hu et al*.*^28^ prepare ZnO nanoparticle-modified magnetic biochar to enhance the biochar adsorption capacity for removing ciprofloxacin. Wu et al.^29^ isolate and immobilize a novel PGPR strain SNB6 on BC as the multiple biochemical materials (BCM) as well as combined with vetiver grass (*Chrysopogon zizanioides* L.) to form BC-PGPR-accumulator system. They cited that the BCM multiple biochemical materials could effectively enhance Cd accumulation of *C. zizanioides* and improve the soil biochemical qualities in Cd highly contaminated soil.

Many researchers have been innovated various methods to adsorb PNP from an aqueous solution such as aluminum metal–organic framework/reduced graphene oxide composite^1^, activated carbon fiber^30^, structured fixed bed with micro fibrous entrapped activated carbon^31^, and microalgal biochar with high adsorption capacity^4^. Wang et al.^32^ prepared a green nano zero-valent iron biochar combined with potassium persulfate for degrading *p*-nitrophenol in water. Using agricultural wastes for treating wastewater could gain double goals for solving the main two environmental problems due to it represents an effective alternative to non-renewable or unsustainable biomass^33^; and water contamination problem. Chen et al*.*^34^ studied the correlation between biochars’ properties, which tend to adsorb and degrade *p*-nitrophenol. It is reported that biochar activated with sulfite enhanced the potential of Cr (VI) detoxification and removal and *p*-nitrophenol (PNP) pollutants^35^. Sugarcane bagasse biochar (SCB) and its Mg activation (Mg-SCB) were investigated to remove ammonium and phosphorus pollutants from synthetic wastewater. The results indicated that the ammonium adsorption from aqueous solutions by both biochar types was highly efficient, equivalent to Charcoal and Zeolite. Successfully, Mg-SCBB had the high ability to remove phosphate from aqueous solutions^36^. However, it is observed that previous studies on the application of Mg-activated biochar for organic contaminant removal did not study PNP's removal efficiency, especially by sugarcane bagasse biochar. Thus, the current work aimed to study the influences of activation of sugarcane bagasse biochar on its characteristics and kinetics for PNP adsorption from wastewater. The optimum conditions for removing PNP were extensively detected to obtain maximum wastewater remediation.

## Results and discussion

### Biochar characterization

#### Effect of Mg activation

The selected physicochemical properties of the sugarcane bagasse (SCBB) and it Mg activation (ASCBB) are shown in (Table 1). Mg activation process make the significant different between physicochemical properties of ASCBB and SCBB and results in Table 1 illustrate such differences. The biochar yield, ash content, pH, H/C molar ratio, O/C molar ratio, Total surface area, Total pore volume and Mean pore diameter of ASCBB were higher than SCBB. Previous studies have shown that the physicochemical properties of biochar, such as pH, surface potential, and surface area, are important factors controlling their environmental applications^37^.

**Table 1:** Physicochemical and surface properties of sugarcane bagasse biochar (SCBB) and Mg-activated sugarcane bagasse biochar (ASCBB) used in the adsorption study.

Parameters	SCBB	ASCBB
Biochar yield, %	36.1	52.12
Volatile gases, %	63.9	47.88
Moisture content, %	9.08	5.07
Ash content, %	14.88	26.61
pH	7.19	9.89
C, %	60.1	51.63
H, %	0.68	0.72
N, %	0.688	0.691
S, %	0.058	0.042
O, %	23.59	30.82
Si, %	0.563	0.501
H/C, molar ratio	0.011	0.014
O/C, molar ratio	0.393	0.597
(O + N)/C, molar ratio	0.404	0.61
CEC, cmol_c_ kg^−1^	86.96	113.02
Total surface area, m^2^. g^−1^	155.41	191.82
Total pore volume p/p^0^, cm^3^ g^−1^	0.13	0.151
Mean pore diameter, nm	2.535	2.567
Zeta potential, mV	− 25.2	− 10.9

#### SEM-image investigation

**Figure 1:**
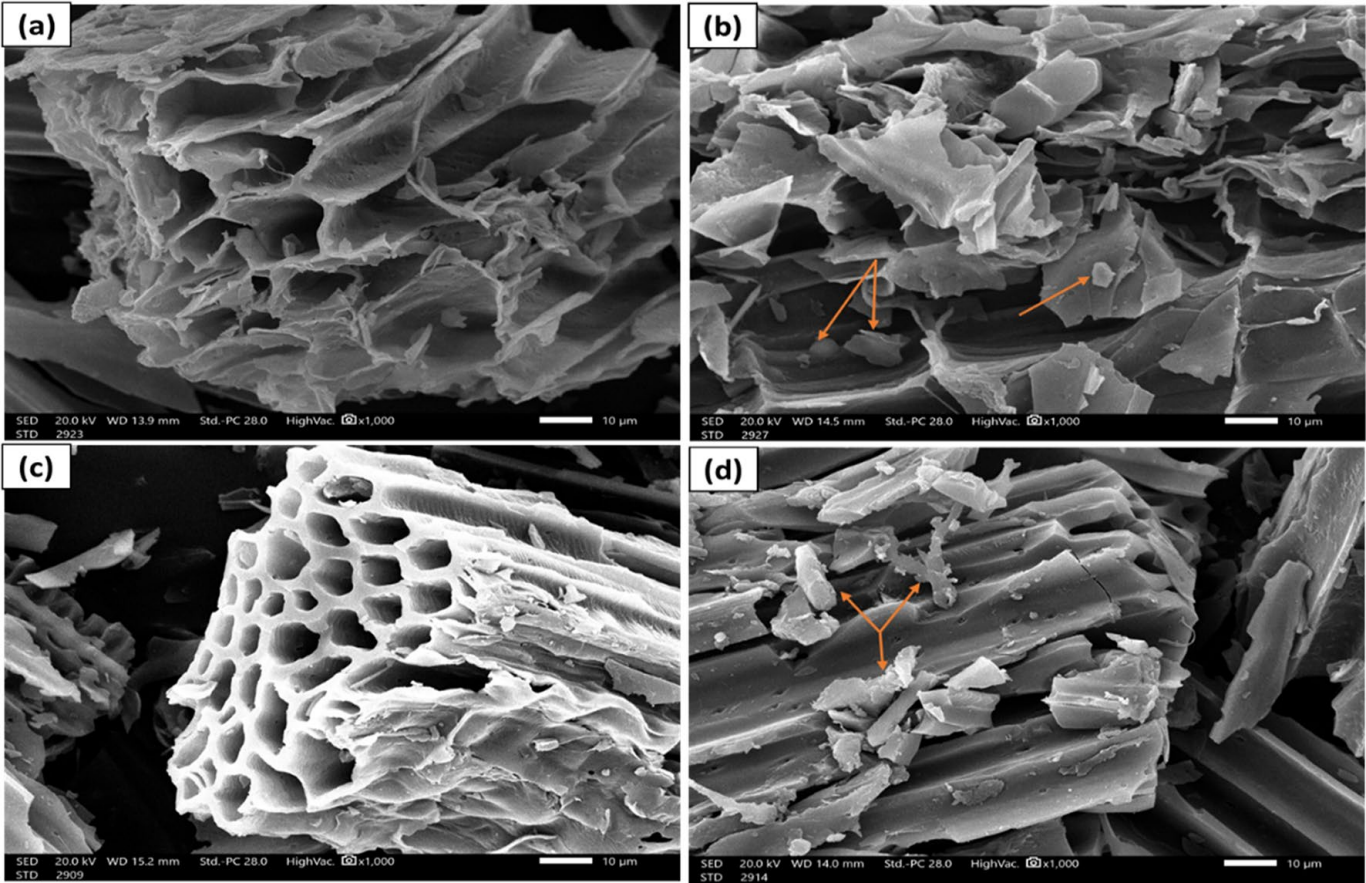
Scanning electron microscopic images of sugarcane bagasse biochar SCBB and its Mg-activation before and after adsorption of 200 mg L^−1^ PNP: (**a**) SCBB before adsorption; (**b**) SCBB after adsorption; (**c**) ASCBB before adsorption; (**d**) ASCBB after adsorption.

The surface morphology of the SCBB and ASCBB before and after PNP adsorption was investigated by scanning electron microscopy (SEM) graphs. The SEM images of both biochar types were shown in (Fig. 1) at a magnification of X1000. The images showed worthy changes to the surface morphology of both biochar types (ASCB and SCBB) before and after the adsorption process (240 min). The SEM images presented various channels in different diameters with a highly complex network of pores for both biochar types, as shown in Fig. 1a,c^38,39^. Notably, the wall boundary structure of ASCBB (Mg-activation, Fig. 1c) became more specific and thicker than SCBB, which indicated that the Mg ions coated the outside and inside of the ASCBB wall structure. In addition, the micropore numbers were augmented in ASCBB biochar compared to SCBB. The total pore volume and mean pore diameter of ASCBB (0.151 cm^3^ g^−1^ 2.567 nm, respectively) are slightly higher than SCBB (0.130 cm^3^ g^−1^ 2.535 nm, respectively), as shown in Table 1. A similar observation was reported by Saleh and Hedia^36^, and Chen et al.^40^. However, the surface pores and channels of SCBB have been blocked after adsorption of 200 mg L^−1^ PNP (Fig. 1b) rather than ASCBB, which appeared the same uniform before adsorption and after 240 min of contact time. This behavior confirmed that Mg-activation supported the biochar wall and increased the surface sorption activity^40^. Notably, for both biochar types, some granular crystals appeared after PNP adsorption on the surface of SCBB (Fig. 1b) and ASCBB (Fig. 1d). The formation of these crystals was higher on the ASCBB surface; the higher the concentration of PNP, the higher the crystal formation. The formation of PNP granular crystals may confirm the adsorption of PNP on the biochar surface, illustrating that maybe it adsorbed PNP on it and blocked the pore channel^36,38,40^.

#### FTIR analysis

**Figure 2:**
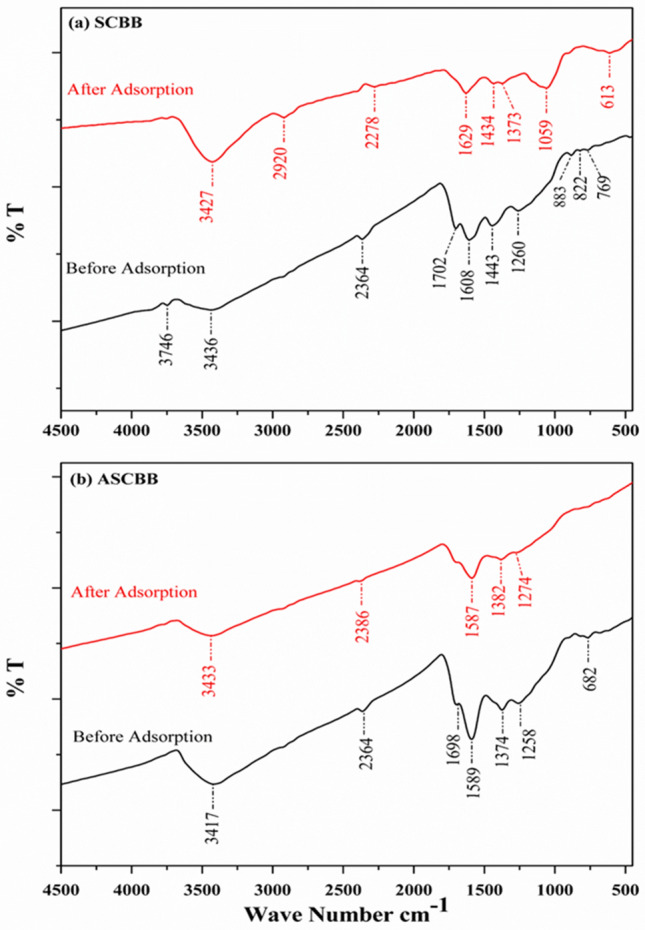
FTIR of the sugarcane bagasse biochar (SCBB, (**a**) and Mg-activation biochar (ASCBB, (**b**) representing the changes in the biochar surface before and after adsorption of PNP (200 mg L^−1^).

Fourier transform infra-red spectroscopy (FTIR) is a vital tool to identify the distinct functional groups, which are instrumental in the adsorption of contaminants. The FTIR spectrum of the two tested biochar revealed many absorption peaks within the interval of 4500–400 cm^−1^, which is only a sign of the complex chemical nature of SCBB. The main functional groups of SCBB and ASCBB are shown in (Fig. 2); the peaks at 3746 cm^−1^ show the existence of a free hydroxyl functional group due to the chemically absorbed water and surface hydroxyl groups. These peaks could be disappeared in the ASCBB before adsorption (Fig. 2b) because of magnesium oxide activation^41^. The peaks around 3436–3433 cm^−1^ for both biochar types corresponding to the O–H caused by carboxylic groups and C–H stretching vibration show alkane functional group. The peak around 2386–2286 cm^−1^ corresponding to the carbonyl, O=C=O bond group, shows alkynes, which became less instance in SCBB after adsorption rather than its original before adsorption. The N–H bend and C=O/C=C stretching vibrations at the peak range 1702–1589 cm^−1^ show amines, alkenes, and aromatic functional groups, which may be attributed to the lignin aromatic groups. The peak around 1400 cm^−1^ shows the aromatic CH and carboxyl-carbonate groups. The bonds of C–O–C of polysaccharides and C–O–H stretching were observed at 1000 cm^−1^. The peaks around 833 to 613 cm^−1^ correspond to C-H stretching aromatic functional, alkyl halides (C–Br stretch) groups and/or the presence of inorganic compounds such as KCl and CaCl_2_^42^. Some of these functional groups shifted after the PNP adsorption process, indicating the surface complex and electrostatic attraction with PNP. The presence of polar groups on the surface is probably going to supply considerable cation exchange capacity to the adsorbent. It is an indication of the active participation of –CO–, –OH, and –C–OH group in PNP binding^37^, whereas the measured values of surface area and cation exchange capacity may indicate an increase in the structural activity between the surfaces or inside the cavities of the biochar nanotubes of sugarcane bagasse biochar.

#### Zeta potential

**Figure 3:**
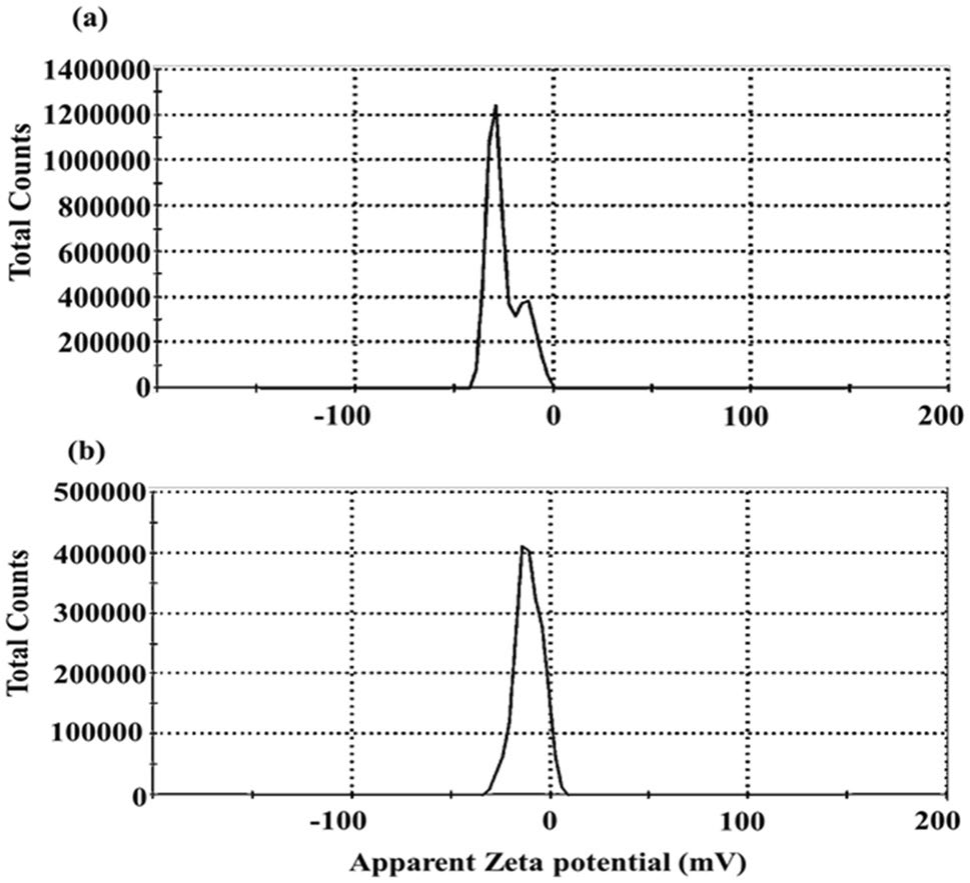
Zeta potential of sugarcane bagasse biochar (SCBB, (**a**) and its Mg-activation biochar (ASCBB, (**b**).

(ζ-potential) is the surface potential associated with the surface electrical charge that affects material particles in suspension. Zeta potential (ζ-potential) is the surface potential associated with the surface electrical charge that affects material particles in suspension like particle complexation, precipitation, surface interaction^43^. Mg-activation significantly influences the ζ-potential of sugarcane bagasse biochar. However, Fig. 3 showed that the negativity surface charge of ASCBB (− 10.9) was decreased relative to SCBB (− 14.3). Interestingly, biochar appears to be effective at remediating cationic pollutants such as heavy metals due to its negative charge. However, it is reported that the stronger the repulsive force (higher colloid stability), the weaker the attraction between nearby particles. It promotes the stability of distributed particles and avoids the formation of aggregates or precipitations^44^. Therefore, compared to ASCBB, SCBB displayed a greater electrostatic interaction with cationic contaminants.

### Batch adsorption kinetic experiments

#### Efficiency of tested biochar for removing PNP from water

**Figure 4:**
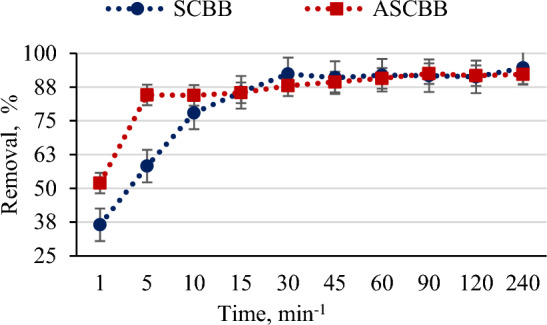
Efficiency of ASCBB and ASCBB for removing PNP (200 mg L^−1^).

**Figure 5:**
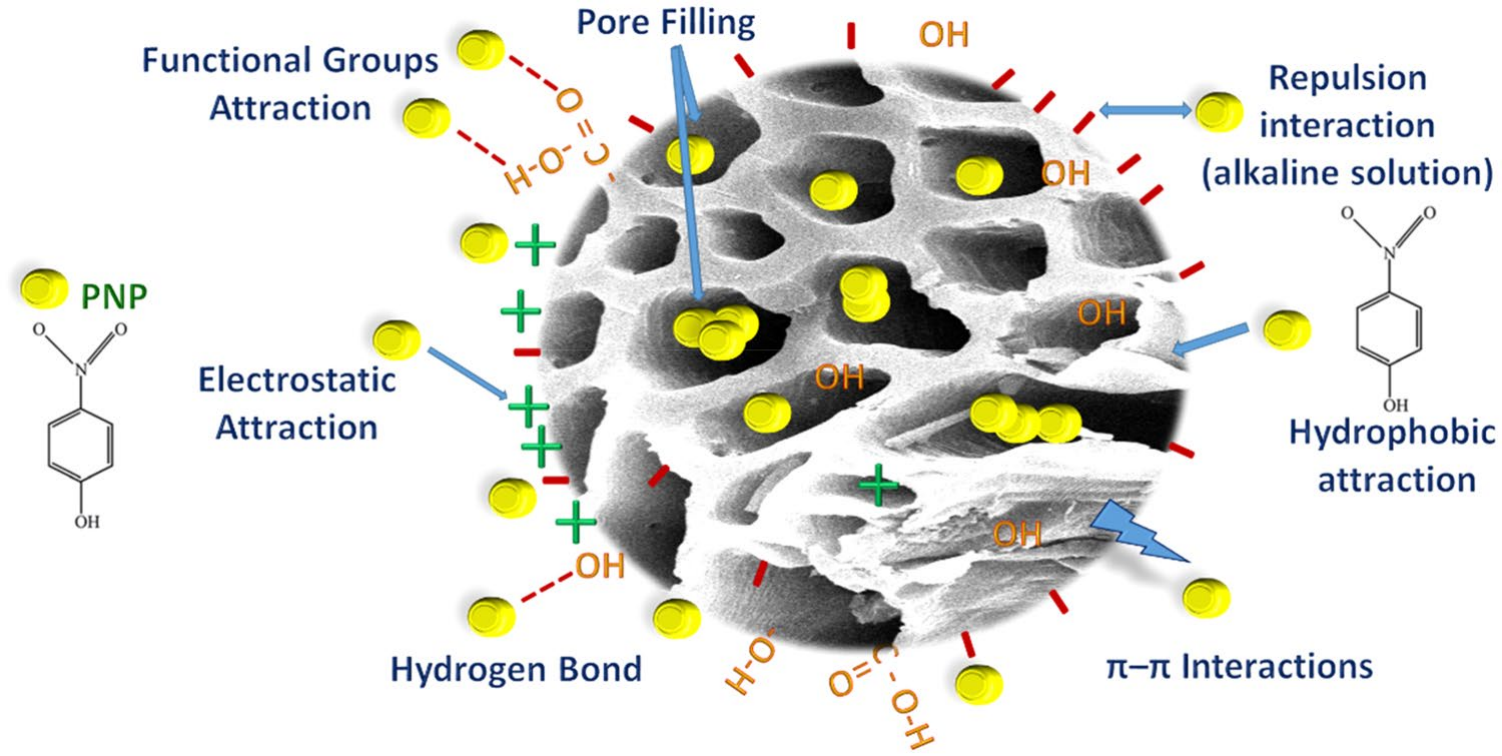
Graphical representation for the possible mechanisms of PNP adsorption on Sugar sugarcane bagasse biochar.

Activated (ASCBB) and non-activated (SCBB) sugarcane bagasse biochar was tested for removing 200 mg L^−1^ of PNP from wastewater in the ratio of 1:100 "W/V" (Fig. 4). The efficiency of ASCBB to remove PNP was 51.98% after 1 min and reached 84.43 in the first 10 min. While, the removal efficiency of SCBB was 36.53% within the first 1 min of reaction time, then raised to 77.94% with increasing time to 10 min. In addition, it was observed that the equilibrium time of the adsorption process of PNP by both investigated biochar types is 15 min (Fig. 4). Also, the adsorption efficiency of both biochar types (ASCBB and SCBB) for removing 50 mg L^−1^ of PNP was evaluated. The data (not shown) clarifies that both investigated biochar types were cable for removing 50 mg L^−1^ of PNP by the same percentage. This result may be due to the high tested biochar (1:100, W/V) relative to a large the amount of PNP (50 mg L^−1^). So, this ratio of PNP-biochar-suspension did not able to make comparison by two suspensions. The fast removal occurred because of sorption developing at the surface, incorporating into porous networks and large surface area that was available for adsorption, which was higher in ASCBB (191.8 m^2^ g^−1^) than SCBB (155.41 m^2^ g^−1^) by about 23%^45–47^. Additionally, CEC value of ASCBB is higher about 1.3 times than SCBB (Table 1). These parameters confirmed ASCBB is more efficient in removing PNP than SCBB. The rapid initial adsorption stage might be because of the hydrophobic interaction between the biochar and the PNP molecules^48^. When the active sites of the biochar surface become fully occupied at equilibrium, the adsorption amount became stable. However, it was reported by Min et al.^49^ that biochar composites (Fe@PP-Hy-Py, 1.0 g L^−1^) have a high ability to remove 90% of 10 mg L^−1^ PNP. In addition, Wang et al.^32^ considered removing 80% of 150 mgL^−1^ PNP using Fe/Zn-biochar in 7 h as fast removing compared to the slower removing extending to 30 h. Moreover, it is estimated that the PNP molecular size was around 0.75 nm, while the mean pore diameter (nm) of both tested biochar materials is higher than PNP molecules by about four times. That means the entire pore surface is available to adsorb more PNP molecules^50^. The pH values of both biochar types are alkaline (Table 1), which means the biochar surface charge is negative due to the ionization of the surface functional groups. Even though these reasons, the surface interaction between PNP and biochar is strong via hydrogen bonding and π–π interactions^27^. From the FTIR study, the formation of new absorption bands, the change in the absorption intensity, and the wave number shift of the functional groups (Fig. 2a,b) could be because of the interaction of the pesticide metabolite with the active sites of the sorbents. However, the rate of adsorption or removal of the metabolite may be more related to the degree of porosity and the biochar structure's internal changes, the activated site's electrostatic attraction with charged molecular groups, or complexation mechanism^27^. Meanwhile, the electron pair sharing between electron donor atoms (O and N) was involved in complexation mechanism. The results obtained in this study suggested that carbonyl, hydroxyl, and amine are the main adsorption sites in SCBB and ASCBB. Figure 5 summarized all possible mechanisms of PNP adsorption on sugarcane bagasse biochar.

#### Effects of pH levels on ASCBB removal efficiency

**Figure 6:**
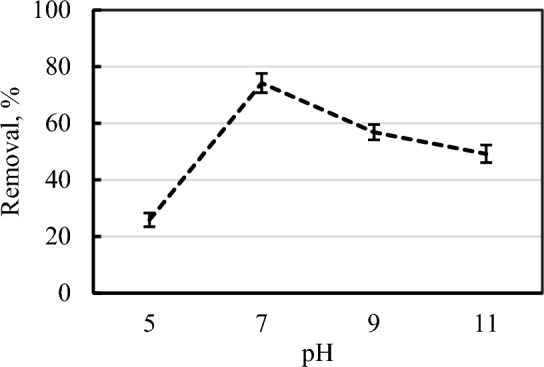
Influence of pH values on the ASCBB removal efficiency for PNP (200 mg L^−1^).

Four levels of pH value (5, 7, 9, and 11) were tested for their influences on 50 mg ASCBB for removing PNP from wastewater. As shown in Fig. 6, the results clearly showed that the adsorption of PNP increased with pH increase at 5–7, and then decreased gradually as pH continuously increased to 11 which was consistent with the result from Wang et al.^50^ who found the PNP adsorption decreased with the increased solution pH. It is reported that the pK_a_ of *p*-nitrophenol is 7.15, so it exists in a neutral species (C_6_H_5_NO_3_); at alkaline media (pH > 7), PNP presence in anionic form (C_6_H_4_NO_3_). It is reported that the pK_a_ of *p*-nitrophenol is 7.15, so it exists in a neutral species (C_6_H_5_NO_3_); at alkaline media (pH > 7), PNP presence in anionic form (C_6_H_4_NO_3_). Additionally, the net surface charge of ASCBB is negative (ζ-potential, − 10.9). That means a sharp decrease of PNP adsorption at high pH condition occurs due to reduction of the electrostatic attraction between PNP ions and ASCBB surface, which led to a weak π–π interaction^51^.

#### Kinetic models

The adsorption kinetic data investigated the potential mechanism of PNP adsorption onto activated (ASCBB) and non-activated (SCBB) biochar samples for initial concentration of 200 mg L^−1^. Five kinetic models were used to evaluate the PNP adsorption technique and the power of sorption rate by ASCBB and SCBB, including Elovich, Power Fraction, Pseudo-first-order, Pseudo-second-order and Intra-particle Diffusion as following:

Elovich kinetic model:

1 $${q}_{t}= \beta ln\left(\alpha \beta \right)+\beta lnt$$

Fractional power kinetic model:

2 $${ln q}_{t}= ln a+b lnt$$

Pseudo-first-order kinetic model:

3 $$ln\left({q}_{e}-{q}_{t}\right)= ln{q}_{e} -{ k}_{1}t$$

Pseudo-second-order kinetic model:

4 $${t/q}_{t}= \frac{1}{{k}_{2}{q}_{e}^{2}}+\frac{1}{{q}_{e}t}$$

Intra-particle diffusion model:

5 $${q}_{t}= {A}_{i}+{K}_{i} {t}^{0.5}$$

where ***q***_***e***_ and ***q***_***t***_ are the sorption capacities (mg g^−1^) of the PNP at the equilibrium and contact time (t, min), respectively, while ***α*** and ***β*** are the initial sorption rate (mg g^−1^ min^−1^) and Elovich constant (g mg^−1^) related to surface coverage and activation energy and ***a*** is the constant (mg g^−1^) and ***b*** is the rate constant (min^−1^) of the power fraction model. ***k***_***1***_ (min^−1^) and ***k***_***2***_ (g mg^−1^ min^−1^) are the constant extent of the Pseudo-first and -second-order sorption models, respectively, and ***k***_***2***_***qe***^***2***^ (h) is the initial sorption rate (mg g^−1^ min^−1^).

***K***_***i***_ (mg min^−0.5^ g^−1^) and ***A***_***i***_ (mg g^−1^) are intra-particle diffusion rate constant and intercept related to the boundary layer thickness at the different stages *i*.

**Figure 7:**
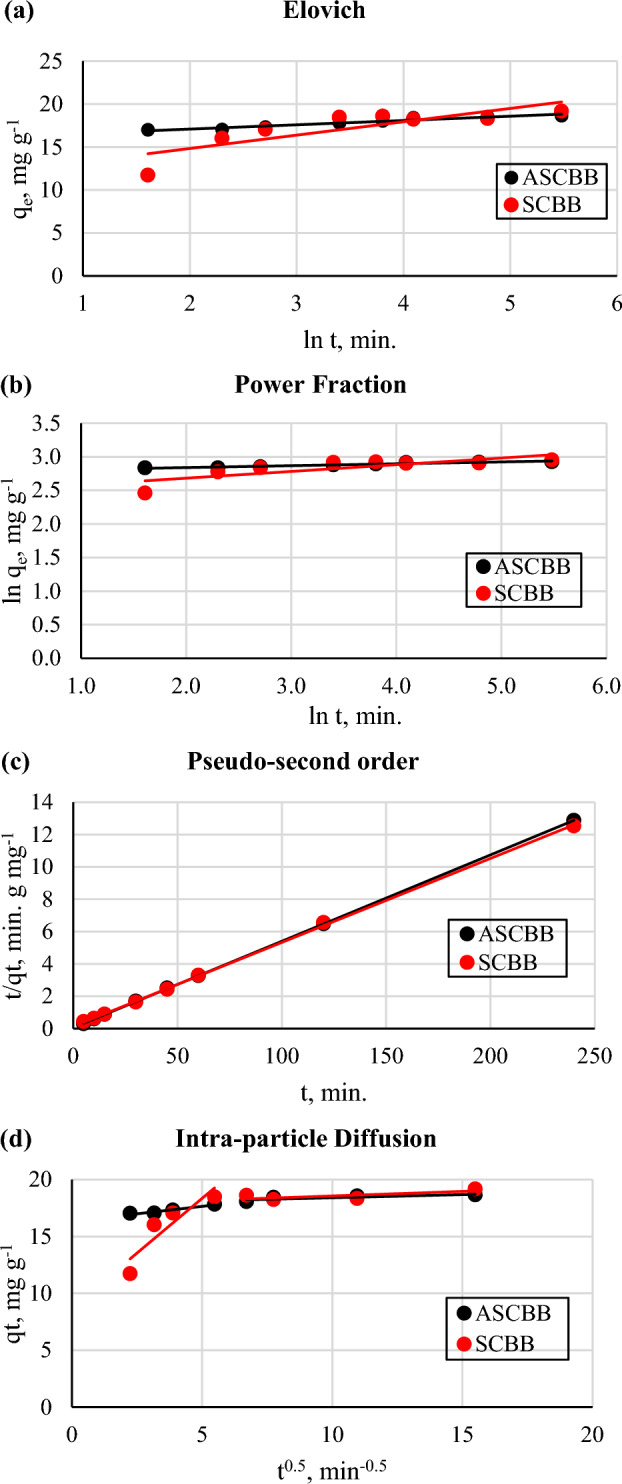
Linear regression of Kinetic models for the adsorption of PNP onto ASCBB and SCBB biochar samples: (**a**) Elovich, (**b**) power fraction, and (**c**) pseudo-second order, and (**d**) intra-particle diffusion.

Figure 7 and Table 2 presented the parameters of examined kinetic equations. In this study, in agreement with kinetic parameters, the Pseudo-first-order model is ineffective in describing the adsorption process of PNP by both biochar types due to the lower calculated R^2^ (coefficients of determination) values 0.0006 and 0.1690 for ASCBB and SCBB, respectively. Another reason is that the model’s calculated sorption capacity (q_e_) values are extremely low compared with experimental q_e_ values (Table 2). The Pseudo-second-order model absolutely recorded the largest R^2^ values 1.00) of the studied kinetic models at the target initial concentrations, showing that the empirical data agree with the pseudo-second-order model. The pseudo-second-order model successfully represented the variations in total PNP sorption capacity and the predicted values of bio-sorption capacity at equilibrium (q_e_) remarkably close to the equilibrium capacities obtained experimentally (Table 2). However, there is no significant effect between the non-activated and Mg-activated sugarcane bagasse biochar on the q_e_ values. The wellness of total PNP sorption to the pseudo-second-order model suggests that the rate-limiting step in the sorption of PNP onto ASCBB and SCBB is chemical sorption^27,52^, which is affected by the active sites (-NH and OH) of the adsorbent at ambient temperature^22^. This model considers that a quick reaction reaches equilibrium fast in the first, followed by a slow reaction that can continue for extended periods for both biochar types^46^. Additionally, these reactions can occur either in sequence or in parallel.

**Table 2:** The constants values of the kinetic models used to interpret the PNP adsorption onto ASCBB and SCBB biochar.

Kinetic model parameters	ASCBB	SCBB
Elovich		
α, mg g^−1^ min^−1^	2.06*10^14^	1209.73
b, g mg^−1^	0.50	1.55
R^2^	0.938	0.685
Power fraction		
a, mg g^−1^	16.17	11.97
b, min^−1^	0.03	0.10
R^2^	0.937	0.642
Pseudo-first order		
q_e_, mg g^−1^	0.425	1.462
K_1_, min^−1^	0.0006	-0.0063
R^2^	0.0006	0.1690
Pseudo-second order		
q_e,_ mg g^−1^	18.73	19.23
h, mg g^−1^ min^−1^	16.50	7.56
K_2,_ g mg^−1^ min^−1^	0.05	0.02
R^2^	1.00	1.00
Intra-particle diffusion		
K_*1*_, mg min^−0.5^ g^−1^	0.26	1.923
A_*1*_, mg g^−1^	16.33	8.73
R^2^	0.934	0.819
K_*2*_, mg min^−0.5^ g^−1^	0.06	0.08
A_*2*_ mg g^−1^	17.85	17.77
R^2^	0.698	0.552

The Elovich model showed a better description of the kinetic data recorded for ASCBB rather than SCBB biochar relative to high R^2^, which was 0.938 for PNP (200 mg L^−1^) adsorbed onto ASCBB. While for SCBB, the R^2^ values was 0.685. Depending on this model, it confirmed that the adsorption process is chemisorption because of irregular energy distribution on the adsorbent surface^22^. The R^2^ values of the power function model were close to Elovich model to describe PNP adsorption by both sugarcane bagasse biochar types (Fig. 7a,b; Table 2). However, biochar type has the main role in the initial sorption rate, meaning thermal or chemical activation causes the change on the active surface site and ions molar ratio (H/C, O/C, and (O + N)/C) of biochar. In this study, we observe that the ion molar ratio of activated biochar is slightly higher than non-activated. However, the interaction of the adsorption mechanism between biochar types and PNP after adsorption was detected due to the shift FTIR spectra (Fig. 2a,b), such as hydrogen bond (3417–3452 cm^−1^), amines, alkenes, and aromatic functional groups in the print finger of sugarcane bagasse (1700–500 cm^−1^). Lui et al.^27^ concluded that the adsorption mechanism of PNP on the bio based porous organic polymers was involved in electrostatic attractions, hydrogen bonding, and π–π stacking interactions. This information was confirmed by studying the surface reactions, zeta potential and FTIR spectra before and after adsorption.

For a better understanding of the major limitations and investigate the impact of mass transfer resistance on the adsorption of *p*-nitrophenol onto SCBB and ASCBB, the intra-particle diffusion model was examined. It is observed that the adsorption process of PNP onto SCBB and ASCBB was divided into two stages depending on the adsorption rate (Fig. 7d). The first one was fast attributed to the process in which *p*-nitrophenol diffuses to the surface of adsorbents. The second one was assigned to intra-particle diffusion through pores after the adsorption process reached the equilibrium stage. According to the results represented in Table 2, the intra-particle diffusion rate constant K_*1*_ was greater than the K_*2*_, which means the removal rate of PNP from the solution in the first stage was higher than that in the second stage for both biochar types that means external diffusion was much faster than internal diffusion^51^. However, the adsorption rate of SCBB in the first stage was higher than that of ASCBB. The intercept (A_*i*_, mg g^−1^) refers to the thickness of the boundary layer, which was higher in the second stage for both tested biochars. Notably, there was no significant difference between A_*1*_ and A_*2*_ for ASCBB that may be due to the Mg activation encourage the surface adsorption and most PNP amount was adsorbed on the ASCBB surface. Moreover, the linear portion of the contact time plot between the PNP and tested biochar (SCBB or ASBB) did not pass through the coordinate origin, implying that the intra-particle diffusion is not the only controlling mechanism of the adsorption process, and the boundary layer diffusion may also influence the adsorption process^46,53,54^.

### Adsorption isotherm

#### Influence of ASCBB doses on PNP removing

**Figure 8:**
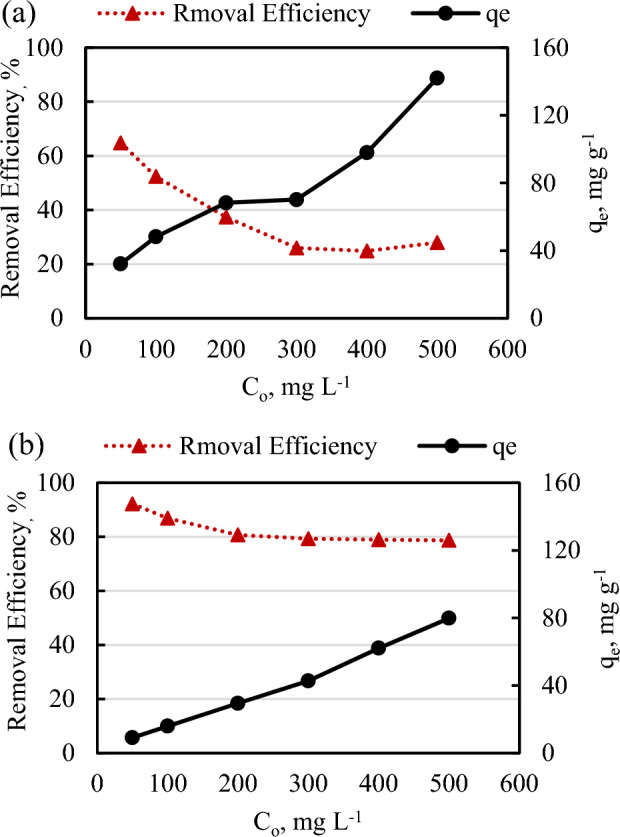
Effect of ASCBB dose on the removal efficiency (%) and adsorption capacity of PNP (50–500 mg L^−1^) via a batch adsorption procedure stirred at 30 °C for 4 h: (**a**) 1 g L^−1^ and (**b**) 5 g L^−1^.

This experiment was designed to detect the role of ASCBB amount (1 g L^−1^ and 5 g L^−1^) for removing series initial concentration of PNP (50–500 mg L^−1^) from synthetic wastewater under batch adsorption experiment extended to 240 min at temperature of 30 °C (pH = 7). As shown in Fig. 8, the results find that the removal efficiency increases with the increasing adsorbent dosage for all PNP-tested concentrations. The removal percentage of 50 mg L^−1^ increased from 65 to 92% when ASCBB dosage increased from 1 to 5 g L^−1^, respectively. However, these percentages decreased to 28% and 79% when the centration increased up to 500 mg L^−1^ for both doses, respectively (Fig. 8a,b). These results were supported by Wang et al.^32^ who reported that when the dosage of green nano zero-valent iron biochar (G-nZVI-BC) increased from 0.2 to 0.8 g L^−1^, the PNP removal rate and reaction rate increased greatly and the reaction rate constant k increased from 0.0306 to 0.06 min^−1^. The increased removal efficiency due to increasing adsorbent dose might be attributed to the rise of adsorbent surface area and its availability of active site numbers to attract PNP from solution. Moreover, this percentage decreased with initial concertation increasing due to the occupied of the adsorbent sites and the accumulation of PNP molecules on the adsorbent surface^55,56^ In contrast of the removal percentages, adsorption capacity (q_e_) of 50 mg L^−1^ adsorbed on ASCBB decrease from 32.20 to 9.16 mg g^−1^ when the ASCBB dosage increases from 1.0 to 5.0 g L^−1^. However, the q_e_ reduction due to increasing adsorbent dose could be attributed to presence of free adsorption sites and increasing sites number availability. Another reason is aggregate formation that leads to decrease the total surface area of adsorbent particle^49^.

#### Adsorption isotherm models of activated biochar

The adsorption isotherm models are used to explain the interaction between adsorbate and adsorbent surfaces. Moreover, these models provide equilibrium information on the adsorbate concentrations in the liquid phase and the amounts adsorbed on the surface of the solid phase^46^.

**Figure 9:**
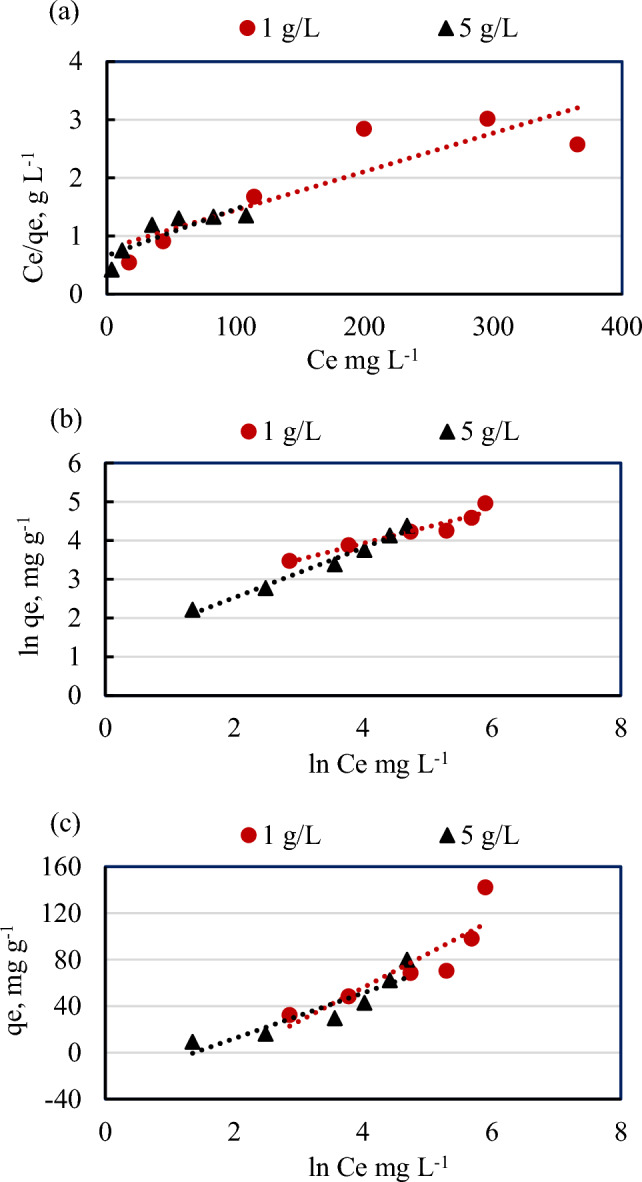
The liner adsorption isotherm models of different concentrations of para-nitrophenol (50–500 mg L^−1^) adsorbent onto two ASCBB dosages (1 and 5 g L^−1^) at 30 °C, agitation time = 240 min, and pH = 7 using Langmuir model (**a**), Freundlich model (**b**) and Temkin model (**c**).

The sorption isotherm parameters describe the relationship between the equilibrium concentrations of PNP (50–500 mg L^−1^) in solutions and the amounts of adsorbed on ASCBB (1 g L^−1^ and 5 g L^−1^), at a constant temperature (30 °C) were illustrate (Fig. 9). For reliable prediction of PNP sorption parameters on the ASCBB, the equilibrium data were modeled with five liner isotherm models of Langmuir, Freundlich, Temkin, Elovich, and Redlich–Peterson (R–P) models as shown as follow:

Langmuir isotherm model

6 $$\frac{{C}_{e}}{{q}_{e}}=\frac{1}{{q}_{max}}{C}_{e}+\frac{1}{{K}_{L} {q}_{max}}$$

Freundlich isotherm model

7 $$\mathrm{ln}{q}_{e}=\frac{1}{n}ln{C}_{e}+ln{K}_{f}$$

Temkin isotherm model

8 $${q}_{e}=B ln{C}_{e}+B ln{K}_{T}$$

Elovich isotherm model

9 $$ln\frac{{q}_{e}}{{C}_{e}}=\frac{1}{{q}_{m}}{q}_{e}+{ln K}_{e} {q}_{m}$$

R–P isotherm model

10 $$\mathrm{ln}\left(\frac{{C}_{e}}{{q}_{e}}\right)=\beta \mathrm{ ln}{C}_{e}-\mathrm{ln}A$$

where, ***q***_***e***_ mg g^−1^) is the PNP adsorbed per gram of ASCBB at equilibrium; ***Ce*** (mg L^−1^) is equilibrium PNP concentration in solution; ***q***_***max***_ (mg g^−1^) is the maximum adsorption capacity of the ASCBB; ***K***_***L***_ (L mg^−1^) is the Langmuir adsorption energy constant. ***K***_***f***_ (mg g^−1^) is the Freundlich constant, and ***n*** the Freundlich exponent. ***B*** and ***K***_***T***_ (g L^−1^) are the Temkins’ model parameters. ***q***_***m***_ (mg g^−1^) and ***k***_***e***_ are the Elovich isotherm constants related to maximium adsorption capacity and initial sorption rate, respectively, whereas ***β*** (L mg^−1^) and ***A*** (L g^−1^) are the Redlich–Peterson isotherm constants.

Langmuir isotherm describes the monolayer formation on a homogeneous surface without interaction between adsorbant and adsorbent surface during the adsorption process, while Freundlich isotherm assumes multilayer adsorption on the adsorbent heterogeneous surface^49^; Temkin's model includes a factor that illustrates adsorbate-adsorbent interactions^51^. Table 3 represented the isotherm parameters of the mentioned models, based on the correlation coefficient (R^2^) values, the investigational data of isotherm models fit better to the Elovich (976–981), R-P (R^2^, 0.956–0.944), and Freundlich (R^2^, 0.921–0.982) than to the Langmuir (R^2^, 0.782–0.723) and Temkin (R^2^, 0.774–0.833) for both dosages. The Freundlich isotherm model implied that the adsorption layer of the PNP onto the ASCBB surface occurred in a heterogeneous multilayer. The adsorption capacity (K_*f*_) of the lower ASCBB dosage (9.39 mg g^−1^), which was greater about 2.71 times relative to the higher dosage (3.46 mg g^−1^). The favorable adsorption of this model can be characterized by the n value. The adsorption is favorable if this value is above 1. In the present study, the n value of both ASCBB dosages is 2.37 and 1.56, which is greater than unity, indicating that the adsorption process is favorable. However, according to the Langmuir parameters, the maximum adsorption capacity (q_m_) values was 151.52 and 125 (mg g^−1^) for 1 and 5 g L^−1^ ASCBB dosages, respectively. In addition, the q_m_ calculated of the lower dosage (q_m, cal_ = 151.52 mg g^−1^) was near to q_e_, _exp_ (142.03 mg g^−1^), which was considerably higher than that calculated of higher ASCBB dosage (*q*_e, cal_ = 125 mg g^−1^; *q*_e_, _exp_ 79.88 mg g^−1^). Therefore, 1 g L^−1^ ASCBB dosage for PNP adsorption is better than 5 g L^−1^. It is concluded that the optimum dosage of KOH activated *Platanus* leaves biochar (PLB) was 2 g L^−1^ to adsorb 300 mg L^−1^ *p*-nitrophenol in a batch experiment conducted at pH = 3, 25 °C, and 150 rpm for 240 min. Moreover, the adsorption capacity of PNP decreased with increasing adsorbent dosage and experiment temperature^52^.

**Table 3:** Langmuir, Freundlich, and Temkin, Elovich and Redlich–Peterson (R–P) isotherm constants of PNP adsorption onto ASCBB.

Isotherm model parameters	1 g L^−1^	5 g L^−1^
Langmuir		
q_*max*_*,* mg g^−1^	151.52	125.00
K_*L*_, L mg^−1^	0.010	0.012
R_*L*_	0.22–0.59	0.19–0.54
R^2^	0.782	0.723
Freundlich		
n	2.37	1.56
K_*f*_, mg L mg^−1^	9.39	3.46
R^2^	0.921	0.982
Temkin		
K_T_, g L^−1^	0.13	0.25
B	29.03	19.57
R^2^	0.774	0.833
Elovich		
q_m_, mg g^−1^	144.93	69.44
K_e_, L mg^−1^	0.007	0.015
R^2^	0.976	0.981
Redlich–Peterson (R-P)		
*A*, L g^−1^	0.11	0.29
*β*, L mg^−1^	0.58	0.36
R^2^	0.956	0.944

The separation factor (R_*L*_) of the Langmuir isotherm essential features can be expressed in terms of a dimensionless constant using the following equation to evaluate the favorability of the PNP adsorption onto the ASCBB.

11 $${R}_{L}=\frac{1}{1+({{K}_{L}C}_{o})}$$

where R_*L*_ (L mg^−1^) is the dimensionless constant separation factor; K_*L*_ (L mg^−1^) is the Langmuir constant related to the energy of adsorption; and C_o_ (mg L^−1^) is PNP the initial concentration.

Generally, the R_*L*_ value is an important parameter related to the Langmuir adsorption isotherm model, indicating the adsorption process's favorability. However, the adsorption is favorable when 0 < R_*L*_ < 1, linear adsorption when R_*L*_ = 1, irreversible when R_*L*_ = 0, and unfavorable adsorption when R_*L*_ > 1^47,57^. Table 3 shows the R_*L*_ values of different initial PNP concentrations for both two dosages of ASCBB. It was observed that the value of R_*L*_ in the range of 0–1 confirms the favorable adsorption of the PNP process (Table 3). Additionally, the dimensionless constant separation factor decreased when initial PNP concentrations increased. That means favorability degree with increasing PNP concentration is irreversibility.

The linear form of Elovich isotherm model descripts multilayer adsorption and the adsorption mechanism is a chemical reaction. The correlation coefficient of Elovich isotherm model recorded the highest value, additionally the maximum adsorption capacity (q_m_) was relatively close with q_e,exp_, which was 144.93 mg g^−1^ for 1 g L^−1^ ASCBB dosage and 69.44 mg g^−1^ for 5 g L^−1^ ASCBB dosage. This model is the best one to explain the interaction between PNP and ASCBB surfaces as compared to the other models.

The R-P isotherm does not exhibit perfect monolayer adsorption behavior because it combines models that describe both homogeneous and heterogeneous systems of the Langmuir and Freundlich equations combination with three parametric approaches^57^. A (L g^−1^) and β represents adsorptive capacity constant and exponent (0 < β < 1), respectively. If β value is close to 1, the model approaches the Langmuir isotherm, and if β value is close to 0, the model approaches the Freundlich isotherm. According to the data represented in Table 3 and Fig. 9e, the β value (0.58–0.36) and with a high R^2^ (0.956–0.944) suggests that this model appears to be useful for representing the equilibrium sorption of PNP by two dosages of ASCBB and the isotherms are nearing Freundlich form predominance of multilayer adsorption^57^.

#### Lab scale cost performance

In Egypt, the cultivated area of sugarcane was about 135.9 thousand hectares that produce annually about 16 million tons^58^. It is estimated that one Ton sugarcane generates about 300 kg of bagasse (wet weight), which is used as fuel in a sugar factory to produce steam and electricity. Additionally, in Egyptian, bagasse could be used for animal feeding, composting, paper, MDF manufacturing, etc. However, the bagasse produced by private juice shops is priceless material and treated as rubbish that causes several environmental problems. Cost estimation of biochar production and its activation is a key consideration from an economic perspective. Therefore, Table 4 summarized the provided lab scale cost performance.

**Table 4:** Lab scale cost estimation.

Material	Amount	Price
Raw material		
Biochar	1 kg	0.35 $
MgSO_4_.7H_2_O (Sigma-Aldrich)	1 kg	318.3
Activation process		
1.6 M (MgSO_4_.7H_2_O) in 100 ml	10 g SCB Feedstock	12.5 $
Activated product	5.2 g Biochar	

## Conclusion

The present study introduces two types of biochar (activated ASCBB and nonactivated SCBB) and evaluated their adsorption potentials for removing para-nitrophenol (PNP) from wastewater. Mg-activation of sugarcane bagasse feedstock provides ASCBB structural and surface characteristics properties with certain privileges better than those of non-activated biochar (SCBB) for using to treat wastewater pollutants. ASCBB provided fast removal of PNP higher than 50% within 1 min. FTIR spectra of polar groups showed that the adsorption mechanism was the electrostatic attractive force, hydrogen-bonding, and π-π interaction. The adsorption of PNP by ASCBB was a chemisorption reaction that confirmed by Pseudo-second-order kinetic model, whereas Elovich, Freundlich and R-P isotherm model confirmed a multilayer adsorption behavior of *p*-nitrophenol on ASCBB surface. Additionally, the separation factor (R_*L*_) value indicating the adsorption of PNP on ASCBB was favorable and its affinity to ASCBB was strong. To conclude, the current study provided the successful waste biomass-derived biochar as a conducive alternative eco-sorbent to eliminate *p*-nitrophenol from wastewater.

## Material and methods

### Feedstock and biochar preparation

Feedstock of sugarcane bagasse (SCB) was collected from a private juice shop and washed several times with tap water followed by distilled water to remove extraneous materials. The clean SCB was cut into smaller particle sizes and dried in oven at 80 °C for 48 h, then divided to two parts and stored in air-tight plastic jars.

#### Activation of sugarcane bagasse (SCB)

For activation process, ten grams of dried SCB were immersed in 100 ml of MgSO_4_.7H_2_O (1.6 M) for 2 h, and then the Mg-saturated feedstock (Mg-SCB) was dried at 80 °C for 48 h^36^.

#### Biochar preparation

The oven-dried feedstocks (activated and non-activated) were converted into biochar through slow pyrolysis at 500 °C for 30 min. under limited conditions of oxygen environment in a muffle furnace (VULCAN A-550). The feedstocks were placed in a ceramic vessel and covered to avoid air contact following the procedures described by Saleh et al.^59^. After the carbonization, the sample was kept in a desiccator and donated as SCBB and ASCBB for non-activated and activated sugarcane bagasse, respectively. The generated biochar samples were ground, sieved using a polypropylene sieve (0.5 mm), and qualified for further tests.

#### Generated biochar characterization

The percentage yield of biochar was calculated according to Eq. (12), based on oven-dry weight of feedstock^39,60^. The volatile gas was calculated by subtracting values of the original mass of feedstock from the generated biochar divided by the original mass of feedstock (Eq. 13). Moisture content was determined based on the mass loss of 5.00 g ± 0.05 of air-dried biochar after dried in the oven adjusted for 24 h at 80 °C as descripted in Eq. (14)^61^.

12 $$\mathrm{Yield }(\mathrm{\%})= \left({W}_{1}/{W}_{0}\right)*100$$

13 $$\text{Volatile \,gas }({\%})= \left({W}_{0}{-W}_{1}/{W}_{0}\right)*100$$

14 $$\text{Moisture \,content }(\mathrm{\%})=\left({{B}_{1}-B}_{2}/{B}_{1}\right)*100$$

where W_0_ (g) is the original weight of the dried feedstock and W_1_ (g) is the weight of the biochar. Whereas, B1 and B2 (g) are the air-dried and oven dried biochar, respectively.

Carbon, hydrogen, nitrogen, and sulphur percentages of both biochar types (ASCBB and SCBB) were determined by CHNS Elemental Analyzer (Vario type, El, elemental analyzer). The oxygen content was obtained according to Liu et al.^22^ (Eq. 15)^62^.

15 $$O \left(\%\right)= 100 -(Ash\% + C\%+ H\% + N\%+S\%)$$

The pH was measured in a cooled biochar/water suspension (1:100, W/V) after stirring for 20 min at 90 °C using pH-meter (Accumet Research AR50 model)^63^. The cation exchange capacity (CEC) of the tested biochar materials was determined using a modified method of ammonium-acetate compulsory displacement^64^. The biochar samples were saturated with CH_3_COONa (1 M, pH 8.2), then the biochar samples were washed with C_2_H_5_OH to eliminate excess Na + from the surface. After that, the CH_3_COONH_4_ solution (1 M, pH 7) was used to displace Na^+^ ions, and the released Na^+^ ion was measured by atomic absorption spectroscopy (each step was repeated three times).

The specific surface area (Bruner-Emmett-Teller method “BET”) was determined using N_2_ adsorption isotherms run on NOVA 1200 instrument at 273 °K.

Scanning electron microscopy (JEOL, Model JSM—IT200, Tokyo, Japan) was used to examine the surface morphology of SCBB and ASCBB before and after PNP adsorption. Before the investigation, the samples were coated with gold using a sputtering coater (JEOL, Model JFC-1100E Ion Sputtering Device) to avoid the buildup of local electrical charges. The SEM instrument was operated at 200 kV/SED; the micrographs were recorded at X1000 magnification scale to characterize the biochar samples' morphology.

Fourier transform infrared (FTIR) spectroscopy analysis was carried out to determine the SCBB and ASCBB surface functional groups using FTIR—6100 JASCO spectrometer in the scanning range 4000–400 cm^−1^.The oven-dried SCBB and ASCBB samples were mixed with potassium bromide powder (KBr, spectroscopic-grade), then ground using agate mortar. 1% (wt) of the finely homogenous mixture was compressed into a disc of 1.2 cm in diameter to obtain a tape have a thickness of about 1 mm. Before FTIR analysis, the KBr disc had been previously scanned as a background.

The surface negative charges of both biochar materials and their particle sizes were determined using Zetasizer (ZP-Malvern-UK) after shaking of biochar-water suspension (0.1 g: 200 ml) at 150 rpm for 12 h^44^.

### Batch adsorption kinetic experiments

#### Influence of activation process on biochar efficiency for removing PNP

An experiment was conducted to determine the performance and efficiency of the two forms of sugarcane bagasse biochar types (SCBB & ASCBB) for removing PNP from artificially contaminated water. A 100 mg of each biochar type was added to a 10 ml solution of PNP (200 mg L^−1^) and the mixture was mechanically stirred for 1, 5, 10, 15, 30, 45, 60, 90, 120, and 240 min at 30 °C. On each interval, the supernatant was filtered immediately before measuring the concentration of PNP in the filtrate using UV–visible Spectrophotometer Alpha 1502 (Laxco, Inc., Bothell, WA 98021, USA) at its maximum absorbance wavelength (410 nm)^50^. The removal efficiency (*R*, %) and adsorption capacity (*q*_*t*_) were calculated from Eqs. (16) and (17), respectively, according to the concentration of PNP in the solution before and after adsorption^4^.

16 $$R =\frac{\left({C}_{i}-{C}_{f}\right)*100}{{C}_{i}}$$

17 $${q}_{t}=\frac{\left({C}_{i}-{C}_{f}\right)*V}{m}$$

where *R* and *q* are the PNP removal efficiency (%) and sorption capacity (mg g^−1^), respectively.* C*_*i*_ and *C*_*f*_ are the initial and final concentrations of PNP in the solution (mg L^−1^), respectively; *V* is the solution volume (L), and *m* is the dry weight of sorbents (g). All measurements were conducted at pH 7 by using phosphate buffer solution pH 7 for adjustment.

#### Effects of pH levels on ASCBB removal efficiency

Effect of pH level on the biochar efficiency for removing PNP removal efficiency by Mg-activated was investigated at different pH levels. The pH of PNP diluted solution was adjusting using 0.1 M NaOH or 0.1 M HCl, through the range of 5–11. Ten millimeters of PNP (200 mg L^−1^) at different pH solution were adding to 50 mL glass flasks with 50 mg (ASCBB). Then, the flasks were sealed and shaken for 1 h before measuring the remaining PNP in solutions as mentioned above. All the tests were performed in triplicates.

#### ASCBB removal efficiency for increasingly PNP concentration

This experiment was carried out to determine the maximum adsorption capacity of two ASCBB dosages (1 g L^−1^ and 5 g L^−1^) using batch experiments. 10 mL of different initial concentrations of PNP solution (50, 100, 200, 300, 400, 500 mg L^−1^) were added into 50 mL glass contains of the tested ASCBB dose. Solutions of PNP were prepared by diluting the initial stock solution using buffer solution (pH = 7). Then, the flasks were sealed and the suspension shaken at 30 °C for 240 min. At the end of the reaction, the remaining concentration of PNP in the suspensions was measured as descripted above.

### Statistical analysis

All presented data were expressed as mean ± standard deviation (SD) of the mean. All data is representative of at least three independent experiments. All statistical analyses were carried out using SPSS version 19 statistical software. Additionally, FTIR peaks were analyzed using OriginPro 8 SRI v8.0773 (B773).

### Ethical approval

The ethical standards were followed precisely during this study. Also, at every stage of the research, authors confirm: (1) No person or animal was exposed to any component of the materials used in the research, so that any harm would occur to him. (2) The authors didn't use any live plants in this investigation. (3) Components or materials were not used in the research in a manner or concentration that would cause direct or indirect harm to the individuals carrying out the research or those in charge of the various measurement processes. (4) All the tools used in the research were dealt with in a scientific, healthy and accurate manner, which entails the safety of individuals and places in accordance with the governing local rules and laws.

## Data Availability

The authors confirm that the data supporting the findings of this study are introduced and available within the manuscript.
